# Adapting for success: T cell features at tissue sites

**DOI:** 10.1093/discim/kyae009

**Published:** 2024-05-18

**Authors:** Philip P Ahern, Emily Gwyer Findlay

**Affiliations:** Department of Cardiovascular & Metabolic Sciences, Lerner Research Institute Cleveland Clinic, Cleveland, OH, USA; School of Biological Sciences, University of Southampton, Southampton, UK

T cells are critical mediators of host health, protecting us from a variety of dangers. As such, they are responders to bacterial and viral infections, they play key tissue-protective and reparative roles, and they maintain homeostatic relationships with a resident microbiome. However, they are also major players in the development of autoimmune and inflammatory diseases such as Multiple Sclerosis and inflammatory bowel disease. Key to all of these processes—both protective and pathological—is the fact that the critical site of T cell action is in tissues such as the gut, lung, skin, and brain, rather than in lymphoid organs. Within these tissues, they face a monumental challenge, needing to remain active to deal with the continual threats posed by invading pathogens and tissue damage, while maintaining peaceful interactions with those microbes whose presence maximizes tissue health. As a consequence, these tissue sites are home to abundant phenotypically and ontogenically distinct populations of T cells that are adapted to deal with this challenge. These populations encompass both unconventional T cells established in tissues early in life, as well as T cells that migrate in from blood or lymph. In both cases, the cells mature *in situ* and develop unique molecular programs that equip them for their unique immunological roles. As a result, tissue-resident T cell populations are often very different from those in lymphoid tissue, and these diverse T cell types play fundamental roles in host physiology and immunity. Understanding how they adopt their unique features in response to tissue-specific stimuli is therefore critical.

In this special issue, we set out to focus solely on T cells at those sites where responses to infection and inflammation commonly occur. This series of papers demonstrates the uniqueness of T cells at barrier sites. In the gut, the lung, the skin, and the reproductive tract, T cells are very different to those in lymphoid tissues and in blood; and they are different between each of these sites.

Firstly, Anna Hipp, Bertram Bengsch, and Anna-Maria Globig [1] describe how IL-17-producing CD8+ T cells develop, and the importance of this to T cell pathology in tissues. These are T cells that have been overlooked compared to their CD4+ equivalent, but which are now known to be important in diseases as diverse as fungal infection, psoriasis, and inflammatory bowel disease. Importantly, the authors summarize current best practices in culturing these cells *ex vivo—*both murine and human populations.

Mahima Swamy, Neema Skariah, and Olivia James [2] describe the intriguing role of IL-15 in the development and maintenance of tissue-resident T cell populations, particularly intraepithelial T cells in the intestine. The fact that intestinal intraepithelial T cells require IL-15 to survive *ex vivo* is a hugely interesting fact that has important implications for the development of therapeutics aimed at T cells at this site.

In concert with this, Sara Alonso and Karen Edelblum [3] discuss the metabolic requirements of these intestinal T cells. They describe how intestinal T cell metabolism differs from T cells in lymphoid tissue, and how it is further altered by contact with the gut microbiome and with pathogenic infections such as *Salmonella* spp.

Kerrie Foyle and Sarah Robertson [4] address the myriad roles played by γδ cells in the reproductive tract, their responsiveness to ovarian steroid hormones, and their potential role in the complications of pregnancy. Moreover, they uncover differences between mouse and human that may relate to particular anatomical differences, highlighting potential limitations associated with overreliance on murine systems, and reinforcing the need for more studies of human T cells at tissue sites.

Damian Maseda, Silvio Manfredo-Vieira, and Aimee Payne [5] present an overview of how T cells in barrier sites, particularly the skin and the gut, interact with the microbial communities at those sites. They draw comparisons across the T cells from different sites and discuss how the wide diversity of T cell populations is essential for effective responses to infection and challenge.

In response to viral infection, T cells produce tissue-specific cytokine patterns. George Finney, Megan MacLeod, and team [6] studied this in the context of influenza infection in the lung. They reveal a layered response, with innate and unconventional T cells providing the initial source of IFN-γ prior to the recruitment of conventional IFN-γ-producing CD4+ and CD8+ cells. These cells localized to the airway parenchyma where they remained primed and ready to respond to subsequent infection, underscoring how the accumulated exposure to pathogens at mucosal tissue sites may shape future responses.

Through a combination of murine and human-based studies, immunologists have uncovered a wealth of information about the mechanisms through which T cells develop at and function in barrier tissue sites. Despite the emerging picture of tissue-specific specialization, the T cell populations we isolate to study in the lab typically originate from the blood or lymphoid tissues such as spleens, and thus have limited capacity to inform on tissue-specific adaptations of human T cells. This collection of papers supports the idea that T-cell responses to inflammatory disease and key physiological events like pregnancy should ideally be studied in the tissue site itself, and even within particular niches within those tissues. This presents a challenge for those of us who work on human immunology, where often only peripheral blood T cells are available. As approaches to deeply phenotype tissue-derived immune populations from humans are advancing, the time is ripe to build on the concepts outlined in this series of articles. Ultimately, this will provide a more complete understanding of the endless and fascinating functions of T cells, provide a framework for comparative immunology, and help to facilitate efforts to shape the functions of T cells in the tissue for therapeutic purposes.
